# Brief Review of Chloroquine and Hydroxychloroquine Toxicity and Management

**DOI:** 10.5811/westjem.2020.5.47810

**Published:** 2020-06-03

**Authors:** Jacob A. Lebin, Kathy T. LeSaint

**Affiliations:** University of California San Francisco, Department of Emergency Medicine, San Francisco, California. California Poison Control System, San Francisco Division, San Francisco, California

## Abstract

As of April 21, 2020, more than 2.5 million cases of coronavirus disease 2019 (COVID-19), caused by the SARS-CoV-2 virus, have been reported in 210 countries and territories, with the death toll at 171,810. Both chloroquine and hydroxychloroquine have gained considerable media attention as possible therapies, resulting in a significant surge in demand. In overdose, both medications can cause severe, potentially life-threatening effects. Here, we present a brief overview of the pharmacology of chloroquine and hydroxychloroquine, manifestations of toxicity, and treatment considerations.

## INTRODUCTION

As of April 21, 2020, more than 2.5 million cases of coronavirus disease 2019 (COVID-19), caused by the SARS-CoV-2 virus, have been reported in 210 countries and territories, with the death toll at 171,810.^1^ While extensive research is underway to evaluate the efficacy of numerous antiviral and other immunomodulatory medications against COVID-19, chloroquine (CQ) and hydroxychloroquine (HCQ), in particular, have gained considerable attention. Data to support the use of CQ and HCQ for COVID-19 are limited and inconclusive, as its use against SARS-CoV-2 has been demonstrated in vitro and in small, poorly controlled, or uncontrolled clinical trials.^2^–^4^

Since being prominently featured in the press as a potential COVID-19 therapy, demand for CQ and HCQ has exploded. On March 31, 2020, the United States Food and Drug Administration added both medications to its drug shortages webpage due to a significant surge in demand.^5^ Soon after, the California Department of Consumer Affairs reported that healthcare providers were wrongfully hoarding and prescribing CQ or HCQ for themselves and family members for COVID-19 prophylaxis despite a lack of evidence to support this use.^6^ In response, several states have issued emergency restrictions on how CQ and HCQ can be dispensed.

Unfortunately, the media attention and the increase in usage of CQ and HCQ do not come without significant consequences. Both medications, when taken in overdose, can cause severe, potentially life-threatening effects. On March 23, 2020, an Arizona man died after an overdose of chloroquine phosphate, formulated as an aquarium cleaner.^7^ In light of recent events, we anticipate emergency departments may see a rise in cases of acute and chronic toxicity from CQ and/or HCQ. Here, we present a brief overview of the pharmacology of CQ and HCQ, manifestations of toxicity, and treatment considerations.

## PHARMACOLOGY & PATHOPHYSIOLOGY

**Figure 1:**
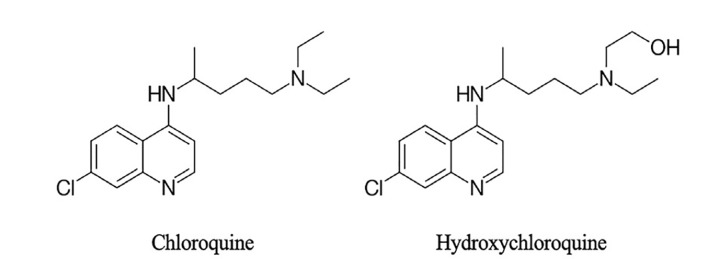
Structural comparison of chloroquine and hydroxychloroquine.^10^

The structurally related compounds CQ and HCQ have historically been used for the prophylaxis or treatment of malaria and share many therapeutic and pharmacokinetic properties. HCQ differs from CQ only by a hydroxyl group, but is considered less potent and is 40% less toxic than CQ in animal models (Figure 1).^8^ Both CQ and HCQ interfere with multiple intracellular processes, resulting in antimalarial, anti-inflammatory, and immunomodulating effects. The anti-inflammatory effects are more pronounced in HCQ, making it useful for the treatment of rheumatoid arthritis and systemic lupus erythematosus.^9^

Oral CQ and HCQ are rapidly and completely absorbed from the gastrointestinal tract with peak whole blood levels reached 1–3 hours after ingestion.^10^ It is this peak level that is responsible for the rapid onset of severe symptoms and most correlates with mortality risk in acute overdose.^11^ Both CQ and HCQ are metabolized by cytochrome (CYP) P450 enzymes. Therefore, concomitant use of CYP2C8 (clopidogrel, gemfibrozil), and CYP3A4/5 (azole antifungals, ciprofloxacin, diltiazem, macrolides, verapamil) inhibitors may raise CQ and HCQ blood levels.^12^ Due to the large volume of distribution and strong tissue-binding properties of these drugs, the terminal half-life is 5–12 days for CQ and 1–2 months for HCQ.^10^ Severe symptoms generally occur over several hours, but patients can have evidence of ongoing toxicity for days following ingestion.

**Figure 2:**
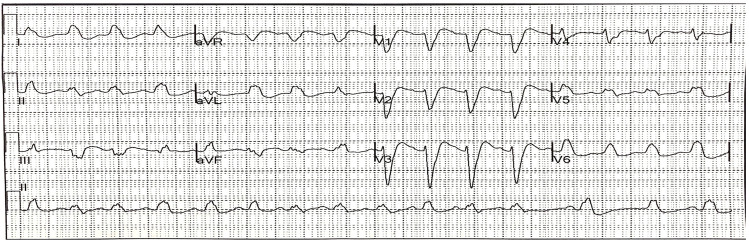
Electrocardiogram from patient with acute hydroxychloroquine overdose demonstrating QRS interval widening (QRS interval = 254 milliseconds).

CQ and HCQ are structural derivatives of quinine and share pathophysiologic mechanisms of toxicity. These drugs have direct cardiovascular toxicity through blockade of voltage-dependent sodium and potassium channels.^13^ This provides the cellular mechanism for the observed QRS and QT interval prolongation (Figure 2). Hypotension and cardiogenic shock are due to direct cardiodepressant effect rather than peripheral vasodilation.^14^ Hypokalemia is common, especially in acute CQ overdoses, and is likely due to an intracellular shift in potassium and not a true, total-body potassium deficit.^15^

## CLINICAL MANIFESTATIONS

CQ and HCQ toxicity is rapid in onset and potentially life-threatening. Severe effects have been associated with ingestion of 5 grams or more of CQ, including systolic blood pressure less than 80 millimeters of mercury, QRS complex duration of 120 milliseconds or more, and hypokalemia (less than 3 millimoles [mmol] per liter [L]). Serum concentrations of CQ greater than 8 micrograms (mcg) per milliliter (mL) have also been associated with severe poisoning, but CQ or HCQ concentrations are unlikely to be readily available during initial assessment and management.^16^ Respiratory depression, central nervous system depression, and seizures have also been described in acute poisoning.^17^

The cardiovascular effects of CQ and HCQ may be precipitous and are frequently the primary cause of mortality. Both drugs act as Vaughan-Williams Class IA antidysrhythmics with “quinidine-like” effect. Electrocardiogram (ECG) changes due to sodium and potassium channel blockade are evident with QRS interval widening, QT prolongation, ST segment depressions, atrioventricular block, and the appearance of U waves.^10^ Cardiac dysrhythmias, including ventricular tachycardia, ventricular fibrillation, and torsade de pointes may result. Hypotension occurs early, is often severe, and progresses rapidly to cardiogenic shock.^17^

**Population Health Research Capsule**

What do we already know about this issue?

*Chloroquine and hydroxychloroquine have gained media attention as possible therapies for coronavirus disease 2019, but both can cause potentially life-threatening effects*.

What was the research question?

*We present an overview of the pharmacology, toxicity, and treatment of chloroquine and hydroxychloroquine overdose*.

What was the major finding of the study?

*Acute toxicity is characterized by direct cardiotoxicity. Supportive care is the mainstay of treatment*.

How does this improve population health?

*Clinicians may see a rise in cases of acute and chronic toxicity from chloroquine and hydroxychloroquine and should be familiar with management strategies*.

Despite severe effects in large, acute ingestions, CQ and HCQ are generally well tolerated at therapeutic doses with mild adverse effects. Common effects are nausea, diarrhea, anorexia, abdominal cramps, rash, and alopecia.^12^ Rarely, sensorineural deafness, visual disturbances, corneal opacities, and irreversible retinopathy can occur with cumulative doses exceeding 100 grams, which usually occurs when CQ and HCQ are dosed as anti-inflammatories.^9^ In addition, agranulocytosis, aplastic anemia, hypersensitivity reactions, hepatitis, myopathy, neuropathy, and cardiomyopathy have been reported with chronic use. CQ and HCQ can act as oxidant stressors, resulting in hemolysis in patients with G6PD deficiency.^10^

## CLINICAL MANAGEMENT

Aggressive symptomatic and supportive care is the mainstay of treatment for both CQ and HCQ overdose. In addition to stabilization of the airway, breathing, and circulation, the patient should receive intravenous (IV) access as well as continuous cardiac monitoring. Serial ECGs should be obtained to monitor for QRS and QT interval prolongation. CQ and HCQ are well absorbed by activated charcoal, and thus should be administered if the risk for aspiration is low. Additionally, given the life-threatening nature of CQ and HCQ poisoning, decontamination with gastric lavage can be considered in cases of a large overdose and if the patient presents soon after ingestion. A medical toxicologist or poison control center should be contacted to assist with management.

Boluses of sodium bicarbonate (1–2 milliequivalents per kilogram [kg] IV) should be provided for QRS interval prolongation to counteract the effects of sodium channel blockade. Of note, the serum alkalinization that results from sodium bicarbonate administration may exacerbate the pre-existing hypokalemia seen from toxicity, which can contribute to further dysrhythmias. However, several reports have suggested that hypokalemia may be protective in severe CQ poisoning.^18^–^20^ Therefore, replacement of potassium is controversial in the setting of acute toxicity, although we believe it would be reasonable to treat severe hypokalemia (i.e., < 2 mmol/L). Cases of rebound hyperkalemia have been reported once toxicity resolves; therefore, serial potassium levels should be obtained.^8^,^19^

Both diazepam and epinephrine have been suggested as specific treatments for CQ and HCQ toxicity. In observational studies, patients with mixed overdoses of diazepam and chloroquine had less toxic effects than those who ingested chloroquine alone.^11^,^19^ Diazepam is believed to decrease CQ and HCQ induced-vasodilation and have central antagonistic, anticonvulsant, and antidysrhythmic effects.^10^ We recommend that patients with severe CQ and HCQ symptoms receive high-dose diazepam therapy (2 milligrams/kg IV over 30 minutes). Because high-dose IV epinephrine (0.25 micrograms/kg per minutes [mcg/kg/min], increasing by 0.25mcg/kg/min until adequate systolic blood pressure) has been the most extensively studied in cases of CQand HCQ-induced hypotension, epinephrine is the vasopressor of choice in these specific ingestions.^16^,^21^ Additionally, combining high-dose diazepam and high-dose epinephrine has shown a potential mortality benefit when compared to controls.^16^ Like sodium bicarbonate, high-dose epinephrine may worsen pre-existing hypokalemia. Finally, a trial of 20% IV fat emulsion (Intralipid) may be indicated in refractory cases, but extracorporeal membrane oxygenation will provide greater benefit if available.^22^,^23^

## SUMMARY

Poisoning from CQ or HCQ can be life-threatening and may become more frequent with increased media attention and use during the COVID-19 pandemic. Acute toxicity is characterized by direct cardiotoxicity, hypokalemia, and precipitous cardiovascular collapse. Treatment includes aggressive gastrointestinal decontamination, sodium bicarbonate for QRS interval widening, and high-dose diazepam and epinephrine in patients with severe toxicity and evidence of shock.
